# Helical flow in tortuous aortas and its relationship to turbulence: A whole-aorta 4D flow MRI study

**DOI:** 10.3389/fcvm.2023.1124604

**Published:** 2023-03-22

**Authors:** Petter Dyverfeldt, Chiara Trenti, Magnus Ziegler, Niclas Bjarnegård, Marcus Lindenberger

**Affiliations:** ^1^ Cardiovascular Sciences; Department of Health, Medicine and Caring Sciences, Linköping University, Linköping, Sweden; ^2^ Center for Medical Image Science and Visualization (CMIV), Linköping University, Linköping, Sweden; ^3^ Department of Cardiology in Linköping, and Department of Health, Medicine and Caring Sciences, Linköping University, Linköping, Sweden

**Keywords:** 4D flow MRI, magnetic resonance imaging, helicity, turbulence, hemodynamics, MR flow imaging, tortousity, ageing

## Abstract

**Background:**

Increased vascular tortuosity is a hallmark of ageing of the vascular system, including the aorta. However, the impact of tortuosity on aortic blood flow is unknown. We hypothesized that increased tortuosity would be associated with increased blood flow helicity and with decreased degree of blood flow turbulence as measured by the turbulent kinetic energy (TKE).

**Methods:**

4D Flow MR images covering the entire aorta from the aortic valve to the iliac bifurcation were acquired in 23 normal volunteers aged 18–30 years (“Young”) and 23 normal volunteers aged 66–76 years (“Old”) without aortic disease. The aorta was segmented and divided into four regions: the ascending, descending, suprarenal abdominal and infrarenal abdominal aorta. Tortuosity, helicity, TKE, flow velocity, and Reynolds number were computed for the whole aorta and for each section.

**Results:**

Tortuosity and helicity were higher whereas TKE, velocity, and Reynolds number were lower in Old than in Young, for all aortic regions (*p *< 0.05) except for helicity in the descending aorta. Tortuosity correlated positively with helicity and negatively with TKE for all aortic regions (Spearman rho=±0.45–±0.72, *p *< =0.002) except for TKE in the ascending aorta. Further, helicity correlated with TKE in the descending, suprarenal abdominal and infrarenal abdominal aorta (Spearman rho=−0.56–−0.77).

**Conclusion:**

Tortuosity increases with age and blood flow in tortuous aortas is more helical. Increasing helicity, in turn, is associated with decreasing TKE.

## Introduction

Age is a major cardiovascular risk factor that contributes to several functional and structural changes to the arterial system. Age-related changes of the aorta include increased stiffness, increased diameter and elongation (1, 2). A particularly visually striking age-related geometric alteration of the aorta is increased tortuosity (1, 3). The mechanism of age-related tortuosity is unclear but has been linked to increased blood pressure and degradation of elastin (4).

Several studies have investigated the association between age-related increase in aortic diameter and blood flow and found that larger aortas have lower flow rates and lower wall shear stress (5–8). Others have explored relationships between the overall shape of the aortic arch and thoracic aortic blood flow and found different secondary flow patterns, such as helical flow, in differently shaped aortas (9). However, impact of tortuosity on aortic blood flow is largely unknown (10).

Blood flow in the normal aorta is well structured and predominately laminar. However, transient velocity fluctuations occur even in healthy individuals and age-related deterioration of the aortic valve appears to cause disturbed flow with elevated velocity fluctuation intensity in the ascending aorta in elderly individuals (11, 12). Similarly, a more tortuous aorta could potentially lead to disturbed flow with increased turbulent velocity fluctuation intensity. Another possible consequence of increased aortic tortuosity is increased flow helicity, which describes the extent to which blood flows in a corkscrew-like patters. The physiological significance of helical flow may be multifactorial as helicity may prevent the accumulation of atherogenic low density lipoproteins on aortic wall, enhance oxygen transport through the wall and reduce the risk of platelet adhesion (13, 14). Interestingly, while turbulent velocity fluctuations are associated with irreversible energy loss and are thereby a hallmark of inefficient blood flow, helical flows are typically laminar. In fact, it has been suggested that the presence of helical flow reduces the negative impact that distorted arterial geometry may have on blood flow efficiency and that this may be caused by natural optimization of efficient blood flow in the cardiovascular system (15, 16). However, previous studies on the phenomenon of helicity-driven flow stabilization in the cardiovascular system have been limited by a lack of methodology to map flow inefficiency in-vivo.

Much of the current understanding of the interrelationship between aortic geometry and blood flow has emerged from studies employing time-resolved, three-dimensional phase-contrast magnetic resonance imaging (4D Flow MRI). 4D Flow MRI provides simultaneously acquired anatomical images and velocity vector maps. The 4D velocity vector maps afford computation of a large number of hemodynamic parameters, including helicity and turbulent velocity fluctuation intensity (17, 18). The co-localized anatomical images obtained with 4D Flow MRI offer several opportunities for investigations of the relationship between vascular blood flow and vascular anatomy. Tortuosity, for example, can be straightforwardly mapped based on a vessel centerline extracted from 3D segmentations of phase-contrast MR angiograms derived by combining the magnitude and velocity vector images (19).

It is well known that age-related alterations of the aorta are not uniform along the aorta. Stiffness, for example, increases more in the proximal than the distal regions of the aorta (20, 21). Similarly, the impact of aortic geometry on blood flow may be different in different regions of the aorta. The hemodynamic environment in the ascending aorta is characterized by jet-like inflow through the aortic valve which dictates the ascending aortic flow environment. Blood flow in the descending and abdominal aorta, on the other hand, resembles pipe flow and may be more affected by aortic geometry. While previous studies on aorta geometry and blood flow have focused on specific subsections of the aorta, we have recently demonstrated the feasibility of whole-aorta 4D Flow MRI from the aortic valve to the iliac bifurcation (22). This extended coverage of 4D Flow MRI enables studies of the relationship between geometry and blood flow in the entire aorta.

The aim of this study was to investigate the relationship between tortuosity, helicity and turbulence intensity in the entire aorta. We hypothesized that increased aortic tortuosity would be related to increased helical flow and that increased helical flow would be associated with less turbulent flow.

## Materials and methods

### Study population

51 subjects were recruited between September 2015 and February 2017 that fulfilled the following criteria: male sex (because the subjects were matched against patients with abdominal aortic aneurysms in a male-only abdominal aorta screening program), absence of aortic disease, sinus rhythm, absence of contraindications for MRI and age either 18–30 years (“Young”, *n *= 25) or 66–76 years (“Old”, *n *= 26). The study was approved by the regional ethical review board and all subjects gave written, informed consent.

### Magnetic resonance imaging

**Figure 1:**
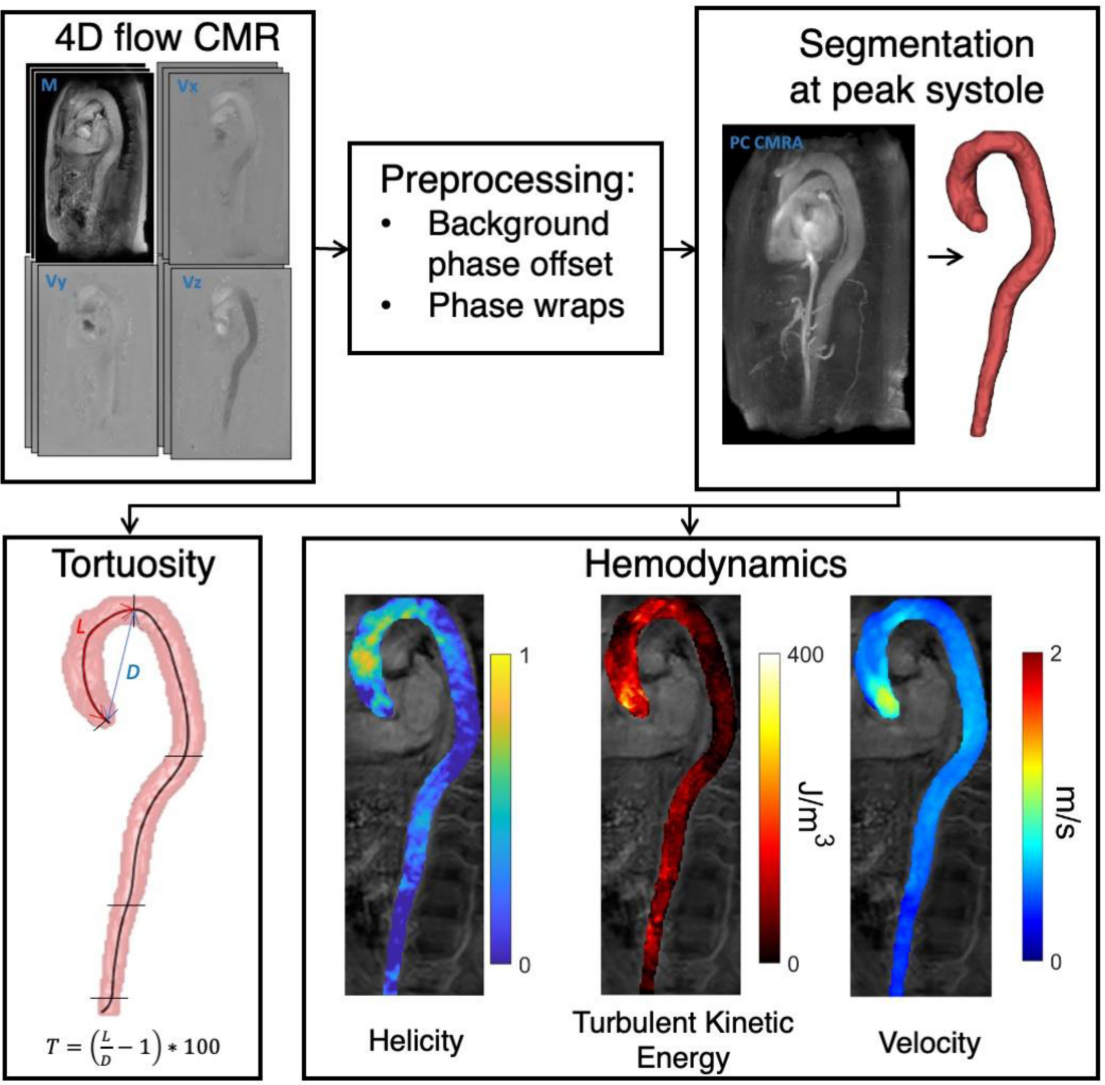
Methods workflow. After preprocessing of 4D flow MRI images, the whole aorta was segmented in PC MRI angiography images. Subsequently, tortuosity (**T**) and the hemodynamic parameters helicity, turbulent kinetic energy, and velocity were computed.

An overview of the methodological workflow is shown in Figure 1. 4D Flow MRI data were acquired using a retrospectively cardiac-gated gradient-echo sequence with four-point asymmetric motion encoding at a 3 T Philips Ingenia scanner (Philips Healthcare, Best, the Netherlands) equipped with a 32-channel torso coil with 60 cm coverage. Data were acquired post injection of a gadolinium-based contrast agent (Magnevist, Bayer Schering Pharma AG). Scan parameters included: VENC=120–200 cm/s, flip angle=15°, echo time=2.5–3.1 ms, repetition time=4.4–5.0 ms, k-space segmentation factor 2. SENSE parallel imaging was used with a total acceleration factor of 4–4.8. The acquired temporal resolution was 35–40 ms and the retrospectively gated data were reconstructed to 40 timeframes using a temporal sliding window-approach. The image volume was adjusted depending on the subject's anatomy to cover the whole aorta from the aortic valve to the iliac bifurcation using a sagittal-oblique slab orientation while maintaining an isotropic voxel size of 2.5 × 2.5 × 2.5 mm^3^. The 3D field of view (FOV) was 480–560 × 480–460 × 71–117 mm^3^ and matrix size was 192–224 × 192–224 × 28–46. Respiratory gating was used with a weighted approach with 6 mm window in the inner 25% and 20 mm in the outer 75% of *k*-space. Total scan time was approximately 10 min including respiratory navigator gating.

4D Flow MRI datasets were corrected for concomitant gradient fields on the scanner, and both phase-wraps and background phase-offset errors were corrected offline. Background phase-offsets were corrected using a weighted 4th order fit to static tissue (23). The magnitude images from the individual flow-encoding segments were reconstructed and used to compute the turbulent kinetic energy (TKE) (17, 24).

### Segmentation

Phase-contrast MRI angiography images were created by combining the 4D Flow magnitude and velocity images and were used to manually create a 3D segmentation the whole aorta with ITK-SNAP (25). Additionally, the centerline along the whole aorta was extracted from the 3D whole-aorta segmentation. An observer (M.L.) with 15 years of experience in aortic MRI positioned landmarks at the aortic valve, the left subclavian artery, the branching of the renal arteries, and the iliac bifurcation in contrast-enhanced MRI angiography images using Horos (horosproject.org). The top of the aortic arch was identified as the most supine centerline point. After transferring these landmarks into the 4D Flow geometry, the 3D segmentation was divided into four regions:

• Ascending aorta (AAo), defined as the region from the aortic valve level to the top of the aortic arch
• Descending aorta (DAo), defined as the region from the top of the aortic arch to midway between the left subclavian artery and the renal artery branches (the diaphragm is not reliably visible in the MR images)
• Suprarenal abdominal aorta (SAA), defined as the region from midway between the left subclavian artery and the renal artery branches to the renal artery branches
• Infrarenal abdominal aorta (IAA), defined as the region from the renal artery branches to the iliac bifurcation

### Tortuosity

The tortuosity of the entire aorta and each subregion were calculated as: $T=\left(\frac{L}{D}-1\right)*100$; where *L* is the centerline length of the segment, and *D* is the Euclidean length from the beginning center point to the end center point of the segment. Tortuosity thus represents the percentage additional length of the aortic region due to tortuosity, when compared to a completely straight aortic segment.

### Hemodynamics

Helicity was quantified by calculating the absolute local normalized helicity: $\mathrm{Helicity}=\left|\frac{V*\omega }{\left|V||\omega \right|}\right|$; where *V* is the velocity field, and *ω* is the vorticity as calculated by ∇×V (16, 26). Helicity is a directionally independent voxel-wise measure where a value of 0 indicates no helical flow, and a value of 1 indicates maximally helical flow. Turbulence intensity was quantified by calculating the volumetric mean TKE in each aortic regions during systole and is denoted TKE (24). TKE per voxel was obtained as $TKE=\frac{1}{2}\rho \overset{3}{\underset{i=1}{\sum }}\sigma_{i}^{2}\left[\frac{J}{m^{3}}\right]$, where ρ is the blood density (1.060 kg/m^3^) and $\sigma_{i}$ is the velocity fluctuation intensity in three orthogonal directions. $\sigma_{i}$ was derived from the magnitude images of the individual flow encodings in 4D flow MRI based on an MR signal model that exploits the effect of pseudorandom fluid motion on the MR signal. For asymmetric four-point motion-encoding, $\sigma_{i}$ is computed as: $\sigma_{i}=\frac{1}{k_{v}}\sqrt{2\frac{\left|S\right|}{\left|S_{i}\right|}}\left[\frac{m}{s}\right]$ where $k_{v}=\pi /VENC$ and |S| and $\left|S_{i}\right|$ are the magnitude of MR signal acquired without and with motion sensitivity respectively (17).

Helicity and TKE were examined at systole for each subject. Peak systole was identified as the peak of the mean velocity profile in each of the aortic regions and data was extracted using a window of ±5% of the cardiac cycle around that time point. In addition to Helicity and TKE, the spatially averaged flow velocity (“Velocity”), average diameter of each segment (“Diameter”) and Reynolds number were extracted for each region. The Reynolds number is used in fluid mechanics to characterize flows in straight pipes, where higher Reynolds numbers are associated with higher likelihood of turbulent flow. The Reynolds number was computed as Re=(ρ∗Velocity∗D)μ, where *ρ* is the density of blood, Velocity is the average flow velocity, D is the average diameter of the segment and *μ* is the viscosity of blood. *ρ* was assumed to be 1,060 [kg m^−3^] and *μ* was assumed to be 3.5e^−3^ [Pa s]. All hemodynamic parameters were computed using MATLAB R2021b (The Mathworks, Natick, MA, United States).

### Statistical analysis

All results are reported as mean ± standard deviation when normally distributed, and median [quartile 1, quartile 3] when not. Two-sample *t*-tests or Mann-Whitney non-parametric tests were used to determine whether or not there were differences between the two cohorts with respect to tortuosity and hemodynamic parameters in each aortic region, after assessing the normality of the data. A significance level (α) of 0.05 was used. Pairwise analyses using the Spearman rank correlation coefficient, *ρ*, were used to examine the relationships amongst hemodynamic parameters and tortuosity. *ρ* was interpreted as follows: ±[0–0.2]: very weak, ±[0.2–0.4]: weak, ±[0.4–0.6]: moderate, ±[0.6–0.8]: strong, and ±[0.8–1]: very strong. MATLAB R2021b was used for statistical analyses. Further, linear regression was used to study the extent to which Helicity and Velocity explains differences in TKE.

## Results

46 subjects were included after exclusions for ECG gating error (*n *= 1), suspension of MRI due to high peripheral nerve stimulation (*n *= 1), and insufficient coverage of the 4D Flow MRI acquisition (*n *= 3). 23 of the subjects belonged to the Young (24 ± 3 y/o) category and 23 to the Old (71 ± 4 y/o). Further demographic and self-reported clinical details relating to the subjects can be found in Table 1.

**Table 1:** Subject demographic and clinical characteristics.

Parameter	Young (*n *= 23)	Old (*n *= 23)	*p*-value
Age [Years]	23 ± 7	71 ± 3	**<0** **.** **001**
Height [cm]	183 ± 6	177 ± 7	**0** **.** **009**
Weight [kg]	78 ± 10	82 ± 8	0.223
BSA [m^2^]	2.0 ± 0.1	2.0 ± 0.1	0.733
SBP [mmHg]	110 ± 7	122 ± 15	**0** **.** **002**
DBP [mmHg]	58 ± 34	72 ± 9	**<0** **.** **001**
Previous cardiac disease	No = 23 Yes = 0	No = 21 Yes = 2*	0.10
Previous lung disease	No = 21 (93%) Yes = 2 (9%)	No = 23 Yes = 0	0.48
Previous cerebrovascular disease	No = 23 (100%) Yes = 0 (0%)	No = 22 Yes = 1	1.0
Previous kidney disease	No = 23 (100%) Yes = 0 (0%)	No = 22 Yes = 1	1.0
Hypertension medication	No = 23 Yes = 0	No = 14 Yes = 9	**0** **.** **001**
Dyslipidemia medication	No = 23 Yes = 0	No = 17 Yes = 6	**0** **.** **02**
Current smoking	No = 23 Yes = 0	No = 23 No = 0	1.00

BSA, body surface area; SBP, systolic blood pressure; DBP, diastolic blood pressure.

* Atrial fibrillation (*n *= 2).

**Figure 2:**
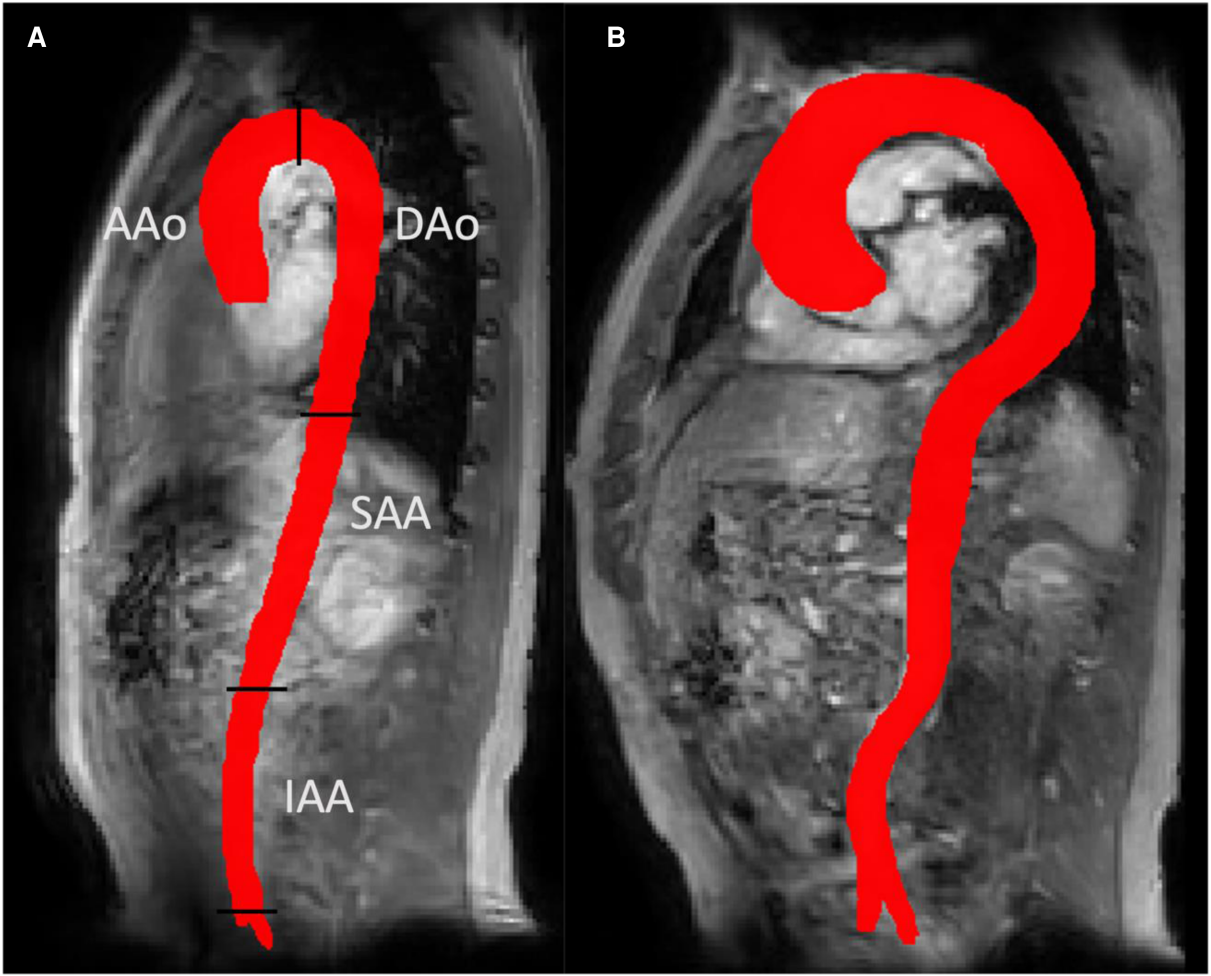
3D aortic segmentations for a representative young subject (panel a) and an old subject (panel b). A more tortuous aorta can be seen in the old subject. Panel a also shows the four aortic segments used for regional analysis in this study: ascending aorta (AAo), descending aorta (DAo), suprarenal and infrarenal abdominal aorta (SAA and IAA).

Figure 2 shows a representative Young and Old aorta. Table 2 shows the cohort-averaged tortuosity and hemodynamics for each region of the aorta. There was a significant difference in tortuosity between the two cohorts throughout the aorta. The largest relative increase in tortuosity between Young and Old was found in the IAA. There were also statistically significant differences between the cohorts for all hemodynamic parameters at all locations, with the exception of Helicity in the DAo. The negative TKE in the IAA in the Old group can be interpreted as a TKE estimate close to zero that appears as negative due to noise.

**Table 2:** Cohort-averaged geometric and hemodynamic parameters per aortic region.

Parameter	Region	Young (*n *= 23)	Old (*n *= 23)	*p*-value
Tortousity [-]	Whole aorta	65 ± 7	99 ± 14	**<0** **.** **001**
	AAo	24 [20, 25]	40 [33, 43]	**<0** **.** **001**
	DAo	12 ± 2	15 ± 4	**0** **.** **002**
	SAA	0.2 [0.2, 0.3]	1.0 [0.5, 1.8]	**<0** **.** **001**
	IAA	0.5 [0.4, 0.7]	2.7 [2.0, 4.1]	**<0** **.** **001**
Diameter [mm]	Whole aorta	18 ± 1	23 ± 2	**<0.001**
	AAo	25 ± 2	30 ± 3	**<0.001**
	DAo	19 ± 1	25 ± 2	**<0.001**
	SAA	16 ± 1	22 ± 2	**<0.001**
	IAA	13 ± 1	16 ± 2	**<0.001**
Helicity [-]	Whole aorta	0.26 ± 0.02	0.31 ± 0.02	**<0.001**
	AAo	0.33 ± 0.02	0.37 ± 0.03	**<0.001**
	DAo	0.25 ± 0.02	0.31 ± 0.03	0.07
	SAA	0.17 ± 0.02	0.26 ± 0.04	**<0.001**
	IAA	0.16 ± 0.03	0.23 ± 0.04	**<0.001**
TKE [J/m^3^]	Whole aorta	38 [31, 45]	18 [11, 23]	**0.002 **
	AAo	44 [36, 51]	35 [21, 51]	**<0.001**
	DAo	40 [30, 46]	8 [0.5, 11]	**<0.001**
	SAA	34 [21, 50]	6 [−8, 14]	**<0.001**
	IAA	20 ± 23	−0.1 ± 13	**<0.001**
Velocity [m/s]	Whole aorta	0.74 ± 0.10	0.43 ± 0.07	**<0.001**
	AAo	0.69 ± 0.12	0.42 ± 0.08	**<0.001**
	DAo	0.78 [0.73 0.87]	0.39 [0.34 0.46]	**<0.001**
	SAA	1.02 ± 0.16	0.49 ± 0.09	**<0.001**
	IAA	0.63 ± 0.09	0.47 ± 0.1	**<0.001**
Reynolds [-]	Whole aorta	3,960 [3697, 4323]	3,020 [2800, 3399]	**<0.001**
	AAo	5137 ± 868	3790 ± 696	**<0.001**
	DAo	4550 ± 640	3054 ± 546	**<0.001**
	SAA	4,824 [4502, 5176]	3,138 [2910, 3623]	**<0.001**
	IAA	2539 ± 334	2264 ± 540	**0.048**

Table 3 lists correlations between the investigated variables. Tortuosity correlates against Helicity, turbulent kinetic energy (TKE) and Velocity with similar strength throughout the aorta. Additionally, Helicity and Velocity were a strongly negatively correlated, and TKE and Velocity were strongly positively correlated. The relationship between Tortuosity and TKE, Tortuosity and Reynolds number, Helicity and TKE, and Helicity and Reynolds number were non-significant in the ascending aorta. Corresponding correlations within the Young and Old groups are listed in Supplementary Tables S1 and S2, respectively.

**Table 3:** List of correlations amongst studied parameters.

Parameters	Region	Rho (interpretation)	*p*-value
Tortuosity—Helicity	Whole aorta	0.86 (very strong)	**<0.001**
	AAo	0.60 (strong)	**<0.001**
	DAo	0.45 (moderate)	**0.002**
	SAA	0.72 (strong)	**<0.001**
	IAA	0.63 (strong)	**<0.001**
Tortuosity—TKE	Whole aorta	−0.63 (strong)	**<0.001**
	AAo	−0.23 (weak)	0.13
	DAo	−0.49 (moderate)	**<0.001**
	SAA	−0.60 (strong)	**<0.001**
	IAA	−0.52 (moderate)	**<0.001**
Tortuosity—Velocity	Whole aorta	−0.75 (strong)	**<0.001**
	AAo	−0.56 (moderate)	**<0.001**
	DAo	−0.44 (moderate)	**0.002**
	SAA	−0.75 (strong)	**<0.001**
	IAA	−0.53 (moderate)	**<0.001**
Tortuosity—Reynolds	Whole aorta	−0.58 (moderate)	**<0.001**
	AAo	−0.45 (moderate)	**0.002**
	DAo	−0.38 (weak)	**0.009**
	SAA	−0.67 (strong)	**<0.001**
	IAA	−0.25 (weak)	0.09
Tortousity—Diameter	Whole aorta	0.80	**<0.001**
	AAo	0.52	**<0.001**
	DAo	0.46	**0.002**
	SAA	0.73	**<0.001**
	IAA	0.53	**<0.001**
Helicity—TKE	Whole aorta	−0.64 (strong)	**<0.001**
	AAo	−0.11 (very weak)	0.5
	DAo	−0.77 (strong)	**<0.001**
	SAA	−0.67 (strong)	**<0.001**
	IAA	−0.56 (moderate)	**<0.001**
Helicity—Velocity	Whole aorta	−0.81 (very strong)	**<0.001**
	AAo	−0.46 (moderate)	**0.002**
	DAo	−0.78 (strong)	**<0.001**
	SAA	−0.78 (strong)	**<0.001**
	IAA	−0.75 (strong)	**<0.001**
Helicity—Reynolds	Whole aorta	−0.61 (strong)	**<0.001**
	AAo	−0.28 (weak)	0.06
	DAo	−0.69 (strong)	**<0.001**
	SAA	−0.68 (strong)	**<0.001**
	IAA	−0.40 (moderate)	**0.006**
Helicity—Diameter	Whole aorta	0.85	**<0.001**
	AAo	0.59	**<0.001**
	DAo	0.81	**<0.001**
	SAA	0.78	**<0.001**
	IAA	0.70	**<0.001**
TKE—Velocity	Whole aorta	0.86 (very strong)	**<0.001**
	AAo	0.56 (moderate)	**<0.001**
	DAo	0.90 (very strong)	**<0.001**
	SAA	0.79 (strong)	**<0.001**
	IAA	0.46 (moderate)	**0.001**
TKE—Reynolds	Whole aorta	0.79 (strong)	**<0.001**
	AAo	0.60 (strong)	**<0.001**
	DAo	0.85 (very strong)	**<0.001**
	SAA	0.77 (strong)	**<0.001**
	IAA	0.27 (weak)	0.07
Velocity—Diameter	Whole aorta	−0.81	**<0.001**
	AAo	−0.80	**<0.001**
	DAo	−0.78	**<0.001**
	SAA	−0.83	**<0.001**
	IAA	−0.61	**<0.001**
Reynolds—Diameter	Whole aorta	−0.50	**<0.001**
	AAo	−0.55	**<0.001**
	DAo	−0.62	**<0.001**
	SAA	−0.65	**<0.001**
	IAA	−0.06	0.7

**Figure 3:**
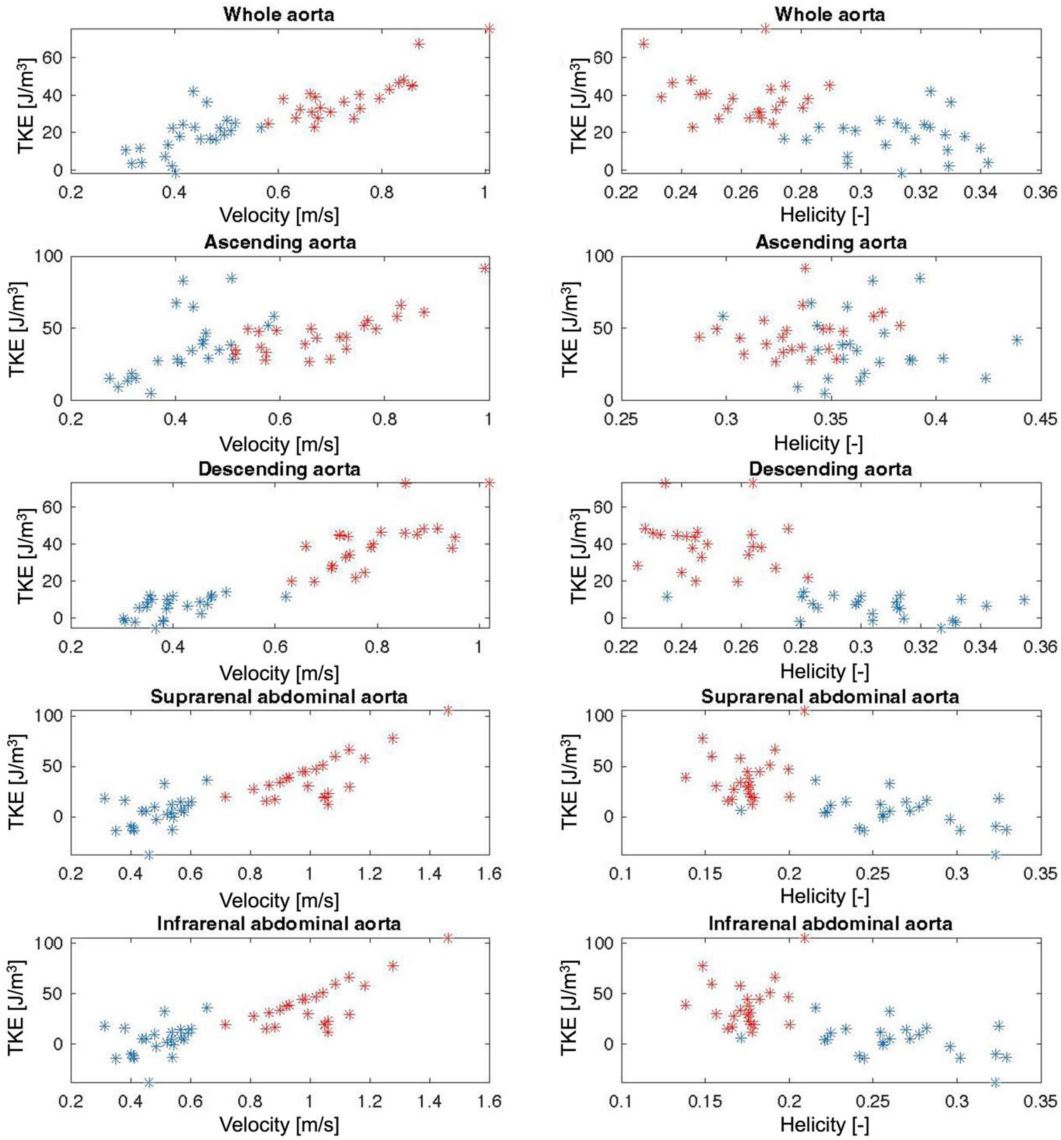
Scatter plots of Velocity and Helicity against turbulent kinetic energy (TKE) for the whole aorta and for each aortic segment. Red stars = Young individuals, blue stars = Old individuals.

Results from the simple and multiple linear regression analyses for the prediction on turbulent kinetic energy (TKE) are shown in Table 4. Simple linear regression showed that Helicity did not significantly predict TKE within the Young group or the Old group, except for in the IAA in the Old group. For the combined study cohort (Young + Old), Helicity significantly predicted TKE in all aortic regions except the AAo. Simple linear regression also showed that Velocity significantly predicted TKE in the AAo, DAo and SAA for both the Young group and the Old group. For the combined study cohort (Young + Old), Velocity significantly predicted TKE in all aortic regions. Multiple linear regression was used to test if Velocity and Helicity predicted TKE in a combined model. Helicity was non-significant in the multiple regression model for all scenarios except for the IAA in the combined study cohort (Young + Old). Velocity significantly contributed to the multiple regression model for most of the aortic regions for Young, Old as well as Young + Old. Scatter plots of the underlying data for Velocity vs. TKE and Helicity vs. TKE are shown in Figure 3.

**Table 4:** Results from simple and multiple linear regression for the prediction of turbulent kinetic energy (TKE) in the aorta.

	Whole	AAo	DAo	SAA	IAA
*Coefficient for Helicity in simple linear regression model*					
Young	−7	−44	−68	−164	−84
Old	−152	140	−153	262	−425
Young + Old	−305	−49	−425	−306	−241
*p-value for coefficient for Helicity in simple linear regression model*					
Young	0.96	0.79	0.16	0.052	0.24
Old	0.36	0.30	0.42	0.34	**0.02**
Young + Old	**<0.001**	0.598	**<0.001**	**<0.001**	**<0.001**
*Coefficient for Velocity in simple linear regression model*					
Young	87	155	41	70	30
Old	100	90	92	106	73
Young + Old	75	63	85	74	77
*p-value for coefficient for Velocity in simple linear regression model*					
Young	**0.004**	**0.002**	**0.008**	0.08	0.25
Old	**<0.001**	**<0.001**	**<0.001**	**<0.001**	0.16
Young + Old	**<0.001**	**<0.001**	**<0.001**	**<0.001**	**0.001**
*Coefficient for Helicity in multiple linear regression model*					
Young	178	69	−16	−116	−53
Old	−63	50	−137	101	−376
Young + Old	102	105	−30	26	−186
*p-value for coefficient for Helicity in multiple linear regression model*					
Young	0.11	0.62	0.74	0.25	0.6
Old	0.52	0.57	0.34	0.62	0.06
Young + Old	0.11	0.21	0.62	0.72	**0**.**04**
*Coefficient for Velocity in multiple linear regression model*					
Young	110	161	39	40	17
Old	99	88	91	104	34
Young + Old	90	71	81	77	26
*p-value for coefficient for Velocity in multiple linear regression model*					
Young	**0.001**	**0.003**	**0.028**	0.39	0.64
Old	**<0.001**	**<0.001**	**0.001**	**<0.001**	0.52
Young + Old	**<0.001**	**<0.001**	**<0.001**	**<0.001**	0.41

AAo, ascending aorta; Dao, descending aorta; SAA, suprarenal abdominal aorta; IAA, infrarenal abdominal aorta.

## Discussion

Ageing brings about several structural and functional changes to the aorta. The development of medical imaging techniques to measure various aspects of aortic structure and function helps to improve our understanding of these age-related changes and thereby establish a ground for understanding aortic disease. Age-related structural changes identified in the present study cohort were increased tortuosity and diameter, and hemodynamic changes were increased flow helicity and decreased flow velocity and TKE. We identified a moderate-to-strong positive correlation between Tortuosity and Helicity, indicating that blood flow in tortuous aortas is more helical. We also identified a moderate-to-strong negative correlation between Helicity and TKE in the descending and abdominal aorta, which demonstrates that helical flows are less turbulent than non-helical flows.

We explored the hypothesis that blood flow in tortuous vessels is more helical, and that the presence of helical flow may suppress flow distortions. This hypothesis was formulated based on previous work by Morbiducci et al. and Gallo et al. (15, 16). In exploring this hypothesis, we investigated relationships between TKE and Helicity. Correlation and simple linear regression analyses demonstrated an inverse relationship between turbulence intensity and helicity, in line with the hypothesis. The relationship between Helicity and TKE, and the role of Velocity which correlated strongly against TKE, was explored further using multiple linear regression. This analysis was done on the complete study cohort as well as the Young and Old groups separately. The significant relationships between Helicity and TKE seen when analyzing the complete study cohort (Young + Old) were in general not seen when analyzing the Young and Old groups separately. We believe that this is due to the homogenous nature of the Young and Old groups. Multiple linear regression with Helicity and Velocity as explanatory variables showed that Velocity was the dominating explanatory variable, and the model was in general not stronger than the linear regression model with Velocity alone. This along with the finding that there is a strong negative correlation between Velocity and Helicity raises the question whether it is the reduction of Velocity, rather than increase in Helicity that results in reduced TKE. However, not only flow velocity but also flow velocity directions and geometrical factors play roles in determining the extent to which arterial flows are helical. For example, it seems likely that increased Helicity, at least in the descending and abdominal aorta, is a consequence of age-related increased tortuosity rather than changes in flow velocity. Moreover, Helicity and Velocity represent different aspects of fluid motion that has separate effects on TKE. Helicity conveys some information about the fluid motion pattern whereas Velocity simply describes how fast the fluid is moving. A non-helical high-velocity flow seems more likely to develop turbulent velocity fluctuations than a corresponding helical flow.

The weakest relationship between tortuosity and helical flow, as well as most other parameter pairings, was found in the AAo. We believe that this regional effect in the AAo is explained by the jet-like flow through the aortic valve. This distinct flow feature has a dominating effect on the hemodynamic environment in the AAo and is a confounding factor for the study of the impact of aortic geometry, which has also been seen in previous studies in ascending aorta hemodynamics (6). A much larger cohort than the one used here may permit stratification based on transvalvular flow velocity and inflow angle and thereby isolate the effects of geometry in the ascending aorta.

The Reynolds number is the ratio of inertial forces to viscous forces within a fluid and is used as an indication of whether flow is laminar or turbulent, but the use of the Reynolds number is limited in arterial flows. The Reynolds number has previously been estimated in aortic flows, but without a reference measure of turbulent velocity fluctuations such as the TKE (27). In idealized situations like steady flow in cylindrical pipes with rigid walls, a Reynolds number of 2,300 marks the border for onset to turbulence. Notably, the Reynolds number as computed in this study was greater than 2,300 in all aortic segments, except in the IAA for Old subjects. However, as the Reynolds number is not intended for flows in distensible tortuous aortas, the use of its actual value is limited. Notably, there was a stronger correlation between Velocity and TKE than Reynolds number and TKE, suggesting that Velocity is a stronger predictor of turbulent flow in the aorta than the Reynolds number. Indeed, reduced Velocity explains the reduces TKE seen in the descending and abdominal aorta in the Old group. The age-related differences in Reynolds number are still of value, but also these may be confounded by the age-related differences in geometry (e.g., tortuosity) and wall stiffness.

### Limitations

A limitation of this study was the relatively limited number of subjects, and the fact that they fall into two relatively distant age brackets. 39% of the individuals in the Old group reported that they had previous or current hypertension, which is higher than what would be expected in a normal population. Additionally, there were no female subjects enrolled in this study. This is because the subjects in this study were originally matched to patients with AAA recruited from a male-only AAA screening program. These cohort-related limitations prevent wider conclusions from being drawn. This study is of cross-sectional nature, preventing any conclusions about causal relationships to be drawn.

## Conclusion

In conclusion, tortuosity increases with age and blood flow in tortuous aortas is more helical. Further, flow velocity and turbulence intensity are in general lower in aortic flows with higher helicity.

## Data Availability

The datasets presented in this article are not readily available because we can only share data in a GDPR compatible way, and within collaborations that meet the criteria of our ethical approval. Requests to access the datasets should be directed to; petter.dyverfeldt@liu.se.

## References

[1] Redheuil A Yu WC Mousseaux E Harouni AA Kachenoura N Wu CO Age-related changes in aortic arch geometry: relationship with proximal aortic function and left ventricular mass and remodeling. J Am Coll Cardiol. (2011) 58:1262–70. 10.1016/j.jacc.2011.06.012 21903061 PMC3508703

[2] Adriaans BP Heuts S Gerretsen S Cheriex EC Vos R Natour E Aortic elongation part I: the normal aortic ageing process. Heart. (2018) 104:1772–7. 10.1136/heartjnl-2017-312866 29593078

[3] Tawfik AM Sobh DM Gadelhak B Sobh HM Batouty NM. The effect of age and gender on tortuosity of the descending thoracic aorta. Eur J Radiol. (2019) 110:54–9. 10.1016/j.ejrad.2018.11.017 30599873

[4] Dobrin PB Schwarcz T Baker W. Mechanisms of arterial and aneurysmal tortuosity. Surgery. (1988) 104:568–71. 3413685

[5] van Ooij P Garcia J Potters WV Malaisrie SC Collins JD Carr JC Age-related changes in aortic 3D blood flow velocities and wall shear stress: implications for the identification of altered hemodynamics in patients with aortic valve disease. J Magn Reson Imaging. (2015) 43:1239–49. 10.1002/jmri.25081 26477691 PMC4836971

[6] van Ooij P Markl M Collins JD Carr JC Rigsby C Bonow RO Aortic valve stenosis alters expression of regional aortic wall shear stress: new insights from a 4-dimensional flow magnetic resonance imaging study of 571 subjects. J Am Heart Assoc. (2017) 6:1–13. 10.1161/JAHA.117.005959 PMC5634265 28903936

[7] Garcia J van der Palen RLF Bollache E Jarvis K Rose MJ Barker AJ Distribution of blood flow velocity in the normal aorta: effect of age and gender. J Magn Reson Imaging. (2018) 47:487–98. 10.1002/jmri.25773 28556277 PMC5702593

[8] Scott MB Huh H van Ooij P Chen V Herrera B Elbaz M Impact of age, sex, and global function on normal aortic hemodynamics. Magn Reson Med. (2020) 84:2088–102. 10.1002/mrm.28250 32162416 PMC7751625

[9] Frydrychowicz A Berger A Del Rio AM Russe MF Bock J Harloff A Interdependencies of aortic arch secondary flow patterns, geometry, and age analysed by 4-dimensional phase contrast magnetic resonance imaging at 3 tesla. Eur Radiol. (2012) 22:1122–30. 10.1007/s00330-011-2353-6 22207269

[10] Han H-C. Twisted blood vessels: symptoms, etiology and biomechanical mechanisms. J Vasc Res. (2012) 49:185–97. 10.1159/000335123 22433458 PMC3369246

[11] Ha H Ziegler M Welander M Bjarnegård N Carlhäll CJ Lindenberger M Age-related vascular changes affect turbulence in aortic blood flow. Front Physiol. (2018) 9:36–36. 10.3389/fphys.2018.00036 29422871 PMC5788974

[12] Stein PD Sabbah HN. Turbulent blood flow in the ascending aorta of humans with normal and diseased aortic valves. Circ Res. (1976) 39:58–65. 10.1161/01.RES.39.1.58 776437

[13] Liu X Sun A Fan Y Deng X. Physiological significance of helical flow in the arterial system and its potential clinical applications. Ann Biomed Eng. (2015) 43:3–15. 10.1007/s10439-014-1097-2 25169424

[14] De Nisco G Kok AM Chiastra C Gallo D Hoogendoorn A Migliavacca F The atheroprotective nature of helical flow in coronary arteries. Ann Biomed Eng. (2019) 47:425–38. 10.1007/s10439-018-02169-x 30488307

[15] Gallo D Steinman DA Bijari PB Morbiducci U. Helical flow in carotid bifurcation as surrogate marker of exposure to disturbed shear. J Biomech. (2012) 45:2398–404. 10.1016/j.jbiomech.2012.07.007 22854207

[16] Morbiducci U Ponzini R Rizzo G Cadioli M Esposito A Montevecchi FM Mechanistic insight into the physiological relevance of helical blood flow in the human aorta: an in vivo study. Biomech Model Mechanobiol. (2011) 10:339–55. 10.1007/s10237-010-0238-2 20652615

[17] Dyverfeldt P Sigfridsson A Kvitting JPE Ebbers T. Quantification of intravoxel velocity standard deviation and turbulence intensity by generalizing phase-contrast MRI. Magn Reson Med. (2006) 56:850–8. 10.1002/mrm.21022 16958074

[18] Morbiducci U Ponzini R Rizzo G Cadioli M Esposito A De Cobelli F In vivo quantification of helical blood flow in human aorta by time-resolved three-dimensional cine phase contrast magnetic resonance imaging. Ann Biomed Eng. (2009) 37:516–31. 10.1007/s10439-008-9609-6 19142728

[19] Ziegler M Alfraeus J Good E Engvall J de Muinck E Dyverfeldt P. Exploring the relationships between hemodynamic stresses in the carotid arteries. Front Cardiovasc Med. (2020) 7:617755–617755. 10.3389/fcvm.2020.617755 33614742 PMC7886794

[20] Taviani V Hickson SS Hardy CJ McEniery CM Patterson AJ Gillard JH Age-related changes of regional pulse wave velocity in the descending aorta using Fourier velocity encoded M-mode. Magn Reson Med. (2011) 65:261–8. 10.1002/mrm.22590 20878761

[21] Dyverfeldt P Ebbers T Länne T. Pulse wave velocity with 4D flow MRI: systematic differences and age-related regional vascular stiffness. Magn Reson Imaging. (2014) 32:1266–71. 10.1016/j.mri.2014.08.021 25171817

[22] Trenti C Ziegler M Bjarnegård N Ebbers T Lindenberger M Dyverfeldt P. Wall shear stress and relative residence time as potential risk factors for abdominal aortic aneurysms in males: a 4D flow cardiovascular magnetic resonance case–control study. J Cardiovasc Magn Reson. (2022) 24:1–12. 10.1186/s12968-022-00848-2 35303893 PMC8932193

[23] Ebbers T Haraldsson H Dyverfeldt P Sigfridsson A Warntjes M Wigström L. Higher order weighted least-squares phase offset correction for improved accuracy in phase-contrast MRI. Proc 16th int'l soc magn reson med. (2008):1367. Toronto.

[24] Dyverfeldt P Kvitting J-PE Sigfridsson A Engvall J Bolger AFAF Ebbers T. Assessment of fluctuating velocities in disturbed cardiovascular blood flow: in vivo feasibility of generalized phase-contrast MRI. J Magn Reson Imaging. (2008) 28:655–63. 10.1002/jmri.21475 18777557

[25] Yushkevich PA Piven J Hazlett HC Smith RG Ho S Gee JC User-guided 3D active contour segmentation of anatomical structures: significantly improved efficiency and reliability. NeuroImage. (2006) 31:1116–28. 10.1016/j.neuroimage.2006.01.015 16545965

[26] Garcia J Barker AJ Collins JD Carr JC Markl M. Volumetric quantification of absolute local normalized helicity in patients with bicuspid aortic valve and aortic dilatation. Magn Reson Med. (2017) 78:689–701. 10.1002/mrm.26387 27539068 PMC5316512

[27] Stalder AF Frydrychowicz A Russe MF Korvink JG Hennig J Li K Assessment of flow instabilities in the healthy aorta using flow-sensitive MRI. J Magn Reson Imaging. (2011) 33:839–46. 10.1002/jmri.22512 21448948

