# Supplementary material for: Helical flow in tortuous aortas and its relationship to turbulence: A whole-aorta 4D flow MRI study
Source: Front Cardiovasc Med. 2023 Mar 22;10:1124604. doi: 10.3389/fcvm.2023.1124604 (PMC10073741; doi:10.3389/fcvm.2023.1124604)
Supplement: Supplementary file 1 [file Table1.docx]

# Supplementary Information

Supplementary Table 1 - List of correlations for the Young group

| **Parameters** | **Region** | **rho** | **p-value** |
| --- | --- | --- | --- |
| Tortuosity - Helicity | Whole aorta | 0.39 | 0.070 |
|  | AAo | -0.001 | 0.998 |
|  | DAo | 0.45 | **0.03** |
|  | SAA | 0.17 | 0.43 |
|  | IAA | 0.60 | **0.003** |
| Tortuosity - TKE_mean_ | Whole aorta | -0.29 | 0.18 |
|  | AAo | -0.2688 | 0.21 |
|  | DAo | -0.004 | 0.99 |
|  | SAA | -0.11 | 0.61 |
|  | IAA | -0.32 | 0.14 |
| Tortuosity - Velocity | Whole aorta | -0.27 | 0.21 |
|  | AAo | -0.27 | 0.21 |
|  | DAo | -0.27 | 0.21 |
|  | SAA | -0.17 | 0.45 |
|  | IAA | -0.41 | 0.052 |
| Tortuosity - Reynolds | Whole aorta | -0.22 | 0.32 |
|  | AAo | -0.35 | 0.10 |
|  | DAo | -0.20 | 0.36 |
|  | SAA | -0.16 | 0.48 |
|  | IAA | -0.33 | 0.12 |
| Tortuosity - Diameter | Whole aorta | 0.29 | 0.18 |
|  | AAo | -0.09 | 0.68 |
|  | DAo | 0.07 | 0.76 |
|  | SAA | 0.05 | 0.84 |
|  | IAA | 0.44 | 0.037 |
| Helicity - TKE | Whole aorta | -0.007 | 0.98 |
|  | AAo | -0.29 | 0.17 |
|  | DAo | -0.27 | 0.21 |
|  | SAA | -0.25 | 0.25 |
|  | IAA | -0.05 | 0.83 |
| Helicity - Velocity | Whole aorta | -0.13 | 0.55 |
|  | AAo | -0.51 | **0.014** |
|  | DAo | -0.60 | **0.003** |
|  | SAA | -0.68 | **<0.001** |
|  | IAA | -0.44 | **0.038** |
| Helicity - Reynolds | Whole aorta | -0.03 | 0.89 |
|  | AAo | -0.36 | **0.09** |
|  | DAo | -0.49 | **0.018** |
|  | SAA | -0.47 | **0.025** |
|  | IAA | -0.27 | 0.21 |
| Helicity - Diameter | Whole aorta | 0.27 | 0.21 |
|  | AAo | 0.39 | 0.069 |
|  | DAo | 0.08 | 0.73 |
|  | SAA | 0.30 | 0.16 |
|  | IAA | 0.42 | **0.046** |
| TKE - Velocity | Whole aorta | 0.68 | **<0.001** |
|  | AAo | 0.56 | **0.006** |
|  | DAo | 0.33 | 0.13 |
|  | SAA | 0.21 | 0.33 |
|  | IAA | 0.65 | **0.001** |
| TKE - Reynolds | Whole aorta | 0.72 | **<0.001** |
|  | AAo | 0.44 | 0.035 |
|  | DAo | 0.35 | **0.01** |
|  | SAA | 0.13 | 0.54 |
|  | IAA | 0.64 | **0.001** |
| TKE - Diameter | Whole aorta | -0.24 | 0.28 |
|  | AAo | -0.14 | 0.53 |
|  | DAo | 0.15 | 0.50 |
|  | SAA | -0.14 | 0.52 |
|  | IAA | -0.20 | 0.36 |
| Velocity – Reynolds | Whole aorta | 0.91 | **<0.001** |
|  | AAo | 0.91 | **<0.001** |
|  | DAo | 0.89 | **<0.001** |
|  | SAA | 0.85 | **<0.001** |
|  | IAA | 0.88 | **<0.001** |
| Velocity – Diameter | Whole aorta | -0.37 | 0.08 |
|  | AAo | -0.19 | 0.37 |
|  | DAo | -0.26 | 0.23 |
|  | SAA | -0.30 | 0.16 |
|  | IAA | -0.38 | 0.07 |
| Reynolds - Diameter | Whole aorta | -0.03 | 0.90 |
|  | AAo | 0.16 | 0.46 |
|  | DAo | 0.08 | 0.72 |
|  | SAA | 0.22 | 0.32 |
|  | IAA | 0.03 | 0.89 |

Supplementary Table 2 - List of correlations for the Old group

| **Parameters** | **Region** | **rho** | **p-value** |
| --- | --- | --- | --- |
| Tortuosity - Helicity | Whole aorta | 0.12 | 0.60 |
|  | AAo | 0.39 | 0.06 |
|  | DAo | 0.12 | 0.57 |
|  | SAA | 0.23 | 0.30 |
|  | IAA | 0.37 | 0.08 |
| Tortuosity - TKE_mean_ | Whole aorta | 0.16 | 0.46 |
|  | AAo | **-0.03** | 0.88 |
|  | DAo | -0.17 | 0.43 |
|  | SAA | -0.10 | 0.64 |
|  | IAA | 0.27 | 0.21 |
| Tortuosity - Velocity | Whole aorta | 0.06 | 0.78 |
|  | AAo | 0.09 | 0.68 |
|  | DAo | -0.26 | 0.23 |
|  | SAA | 0.03 | 0.88 |
|  | IAA | 0.20 | 0.37 |
| Tortuosity - Reynolds | Whole aorta | 0.09 | 0.69 |
|  | AAo | 0.34 | 0.11 |
|  | DAo | 0.04 | 0.86 |
|  | SAA | 0.14 | 0.52 |
|  | IAA | 0.26 | 0.23 |
| Tortuosity - Diameter | Whole aorta | -0.10 | 0.66 |
|  | AAo | 0.50 | **0.016** |
|  | DAo | 0.30 | 0.17 |
|  | SAA | 0.04 | 0.85 |
|  | IAA | 0.08 | 0.71 |
| Helicity - TKE | Whole aorta | 0.30 | 0.17 |
|  | AAo | -0.20 | 0.35 |
|  | DAo | 0.07 | 0.76 |
|  | SAA | -0.40 | 0.06 |
|  | IAA | -0.13 | 0.56 |
| Helicity - Velocity | Whole aorta | 0.15 | 0.50 |
|  | AAo | -0.01 | 0.96 |
|  | DAo | 0.12 | 0.57 |
|  | SAA | -0.43 | 0.04 |
|  | IAA | -0.17 | 0.44 |
| Helicity - Reynolds | Whole aorta | 0.29 | 0.18 |
|  | AAo | 0.08 | 0.73 |
|  | DAo | 0.30 | 0.17 |
|  | SAA | -0.07 | 0.75 |
|  | IAA | 0.09 | 0.69 |
| Helicity - Diameter | Whole aorta | 0.32 | 0.14 |
|  | AAo | 0.34 | 0.11 |
|  | DAo | 0.30 | 0.16 |
|  | SAA | 0.50 | **0.02** |
|  | IAA | 0.44 | **0.04** |
| TKE - Velocity | Whole aorta | 0.66 | **<0.001** |
|  | AAo | 0.66 | **<0.001** |
|  | DAo | 0.52 | **0.013** |
|  | SAA | 0.34 | 0.12 |
|  | IAA | 0.75 | **<0.001** |
| TKE - Reynolds | Whole aorta | 0.67 | **<0.001** |
|  | AAo | 0.62 | **0.002** |
|  | DAo | 0.50 | **0.016** |
|  | SAA | 0.21 | 0.32 |
|  | IAA | 0.74 | **<0.001** |
| TKE - Diameter | Whole aorta | -0.13 | 0.55 |
|  | AAo | 0.08 | 0.73 |
|  | DAo | -0.11 | 0.63 |
|  | SAA | -0.08 | 0.72 |
|  | IAA | 0.10 | 0.66 |
| Velocity – Reynolds | Whole aorta | 0.91 | **<0.001** |
|  | AAo | 0.87 | **<0.001** |
|  | DAo | 0.85 | **<0.001** |
|  | SAA | 0.81 | **<0.001** |
|  | IAA | 0.85 | **<0.001** |
| Velocity – Diameter | Whole aorta | -0.39 | 0.07 |
|  | AAo | -0.11 | 0.62 |
|  | DAo | -0.38 | 0.07 |
|  | SAA | -0.47 | **0.026** |
|  | IAA | -0.10 | 0.65 |
| Reynolds - Diameter | Whole aorta | -0.12 | 0.59 |
|  | AAo | 0.31 | 0.14 |
|  | DAo | 0.09 | 0.68 |
|  | SAA | 0.04 | 0.84 |
|  | IAA | 0.31 | 0.15 |
